# Representation of tree cover in global land cover products**:** Finland as a case study area

**DOI:** 10.1007/s10661-021-08898-2

**Published:** 2021-02-12

**Authors:** Titta Majasalmi, Miina Rautiainen

**Affiliations:** 1 School of Engineering, Department of Built Environment, Aalto University, P.O. Box 14100, 00076 Aalto, Finland; 2 School of Electrical Engineering, Department of Electronics and Nanoengineering, Aalto University, P.O. Box 14100, 00076 Aalto, Finland

**Keywords:** Boreal, Canopy cover, Forest cover, MODIS, CCI LC, MS-NFI

## Abstract

Forest extent mapping is required for climate modeling and monitoring changes in ecosystem state. Different global land cover (LC) products employ simple tree cover (referred also as “forest cover” or even “vegetation cover”) definitions to differentiate forests from non-forests. Since 1990, a large number of forest extent maps have become available. Although many studies have compared forest extent data, they often use old data (i.e., around the year 2000). In this study, we assessed tree cover representations of three different annual, global LC products (MODIS VCF (MOD44B, Collection 6 (C6)), MCD12Q1 (C6), and CCI LC (v.2.1.1)) using the Finnish Multi-Source National Forest Inventory (MS-NFI) data for the year 2017. In addition, we present an intercomparison approach for analyzing spatial representations of coniferous and deciduous species. Intercomparison of different LC products is often overlooked due to challenges involved in non-standard and overlapping LC class definitions. Global LC products are used for monitoring changes in land use and land cover and modeling of surface fluxes. Given that LC is a major driver of global change through modifiers such as land surface albedo, more attention should be paid to spatial mapping of coniferous and deciduous species. Our results show that tree cover was either overestimated or underestimated depending on the LC product, and classification accuracy varied between 42 and 75%. Intercomparison of the LC products showed large differences in conifer and deciduous species spatial distributions. Spatial mapping of coniferous and deciduous tree covers was the best represented by the CCI LC product as compared with the reference MS-NFI data.

## Introduction

Forest extent mapping is required for land cover (LC) and land use classification, and for monitoring changes (e.g., damage, afforestation, deforestation) in ecosystem state. In addition, many land surface model (LSM) components of climate models, which simulate surface fluxes of momentum, heat, and moisture, need a land cover description. The success of classifying forest areas using a satellite image depends on the applied definition and estimation accuracy at a pixel level. Since 1990, a large number of forest extent maps have become available for different purposes at a spatial ground resolution ranging from 300 meters to 5 kilometers (e.g., Bartholome and Belward 2005; Friedl et al. 2002; Hansen et al. 2003, 2000; Loveland et al. 2000; Poulter et al. 2015).

The quantification of global forest resources spatially and temporally relies on the international definition of forest land area by the Food and Agriculture Organization of the United Nations (FAO): a forest is “land spanning more than 0.5 hectares with trees higher than 5 meters and a canopy cover of more than 10 percent, or trees able to reach these thresholds in situ.” (FRA 2015). The canopy cover (CC) is defined as the proportion of ground covered by vertical tree crown projection (Jennings et al. 1999). In practice, directly applying the FAO forest definition in remote sensing remains challenging due to an overlap in LC class definitions. For example, forest area based on the FAO definition does not include land that is predominantly under agricultural or urban land use (FRA 2015) even if the forest definition requirements would be satisfied. Due to challenges in implementing the forest definition by FAO, different global LC products employ simple CC (referred also as “tree cover”, “forest cover,” or even “vegetation cover”) definitions to differentiate forests from non-forests. For example, the International Geosphere-Biosphere Programme (IGBP) employs a tree cover threshold of >60%, and the United Nations Framework Convention on Climate Change (UNFCCC) uses tree cover thresholds of >10% (e.g., FAO international forest definition) and >30% (e.g., FAO global forest ecological zone mapping) to classify an area as a forest. The European Space Agency Climate Change Initiative’s Land Cover (ESA CCI LC) 1992–2015 map series has been developed to provide a complete surface representation for global-scale modeling studies and employs a tree cover threshold of >15% (which has two further sub-classes: 15–40% and >40%). The latest release of the global CCI-LC products covers years 2016, 2017, and 2018. In addition, the binary “forest” or “non-forest” classification, such as that employed by the FAO, has been found to be insufficient on many occasions, and thus, there is an ongoing process of moving towards mapping tree cover as a continuous field (e.g., Sexton et al. 2016). For instance, an operational vegetation continuous field (VCF) product (MODIS VCF 2017) from MODIS (i.e., MOD44B) is known to suffer from underestimating high cover and overestimating low cover (e.g., Heiskanen 2008; Sexton et al. 2013).

There are many definitions of “tree cover” to suit different needs. The definition of tree crown cover accounts for within-crown gaps as a part of the crown, and is measured in the vertical direction without double-counting of overlapping crown projection areas (e.g., Gschwantner et al. 2009). Alternative definitions, which are sometimes used interchangeably, such as canopy closure (i.e., the fraction of hemispherical sky visibility at one single spot on the ground (Jennings et al. 1999)) or effective canopy cover (i.e., takes into account both gaps between crowns and gaps within crowns (Rautiainen et al. 2005)) also exist. The difference between definitions is related to the context in which they are used: the effective CC is preferred when estimating ecological variables such as fraction of absorbed photosynthetically active radiation (fPAR) or leaf area index (e.g., Chen et al. 1997; Gower et al. 1999), while the CC is mainly used to map forest extent and dynamics (e.g., Poulter et al. 2015). Ignoring whether a tree crown contains small gaps or not (i.e., application of different definitions) results in systematic biases. However, it is often not easy to determine which exact tree cover definition the satellite-based LC products are based on. Thus, for simplicity, “tree cover” is used in this paper to refer to all tree cover definitions employed by different LC products.

The increasing importance of spatially explicit data on forest extent mapping is highlighted by recent papers such as Bastin et al. (2019). They mapped the global potential of increasing tree cover to mitigate climate change and concluded that at least 900 million hectares of forest could be restored under current climate conditions. Yet, their conclusions rely on existing tree cover maps, that is, the potential tree cover was obtained after subtracting the current tree cover from a tree cover map produced by Hansen et al. (2013). This demonstrates the necessity of accurate initial mapping of forest extents and properties. Errors in initial mapping and ambiguities in the employed forest areal definition can be expected to transfer to the results and conclusions of follow-up studies utilizing the LC product. In addition, although many recent studies comparing different products exist, they are using old data, such as from years 1992–2010 (e.g., Tang et al. 2019; Song et al. 2014), and rarely consider the impacts of employed classification on spatial patterns of conifer and deciduous tree covers.

In any boreal region, the separation between coniferous and deciduous species groups is essential (e.g., to characterize seasonal courses of surface fluxes in a climate model). This has been recognized and currently, all global LC products differentiate between forests belonging to different phenological groups, such as evergreen needleleaf forest (ENF) and deciduous broadleaf forest (DBF). However, as all LC products employ their own class labeling systems and different tree cover thresholds, validation and intercomparison of conifer and deciduous tree cover representations of different LC products have remained complicated.

Validation of low and medium spatial resolution tree cover products is challenging with traditional field inventories, and thus, higher spatial resolution optical satellite data based products provide the best basis for assessing and evaluating different medium spatial resolution LC classifications. In general, the preference for finer spatial resolution is supported by the smaller fraction of mixed pixels, that is, containing other LC types such as built environment or grassland. Furthermore, in some countries, there already exist nationwide multi-source (MS) thematic maps produced by National Forest Inventories (NFIs). These maps quantify local and regional forest characteristics with a high spatial resolution, at least when working in a global LSM context. The concepts of low, medium, and high spatial resolution are highly dependent on the discipline they are used in. In this study, low spatial resolution refers to spatial resolution commonly applied in LSM at 0.05° (i.e., approximately 5.6 km), whereas medium and high spatial resolutions denote, for example, 250–500 m, and 16-m map products, respectively.

In boreal Finland, systematic monitoring and classification of forested areas have been conducted since the 1920s, in regular 5–10-year cycles (Tomppo et al. 2010). The NFI was developed to produce information on regional forest resources (i.e., stem volume, growth and quality of growing stock, and forest carbon stocks and their changes, forest health, biodiversity of forests, land use structure, and forest ownership), and satellite images have been used in NFIs since the 1980s. Finnish forests are classified into groups of forest land, poorly productive forest land, and unproductive land depending on forest wood production capability. All Finnish boreal forest is secondary forest (i.e., no pristine forest exists) and under active forest management. Forest floor understory species have been inventoried using a nationwide sampling grid three times (in the 1950s, 1980s, and 1995), and the fourth round will take place between 2021 and 2022 (LUKE 2020). The high-resolution MS-NFI maps are produced based on data from NFI field plots, remotely sensed data, and other information sources by national forest authorities and thus provide the best local estimate on the forest characteristics. They also carry information regarding the share of deciduous species and can be used to map geographical variations in land surface phenology (Moon et al. 2019).

In LSMs, LC products define the spatial locations of different vegetation types and often employ what is called tile-based approaches (or “subgrid of vegetation cover” or “fractional cover”). In other words, for each LC unit, information on cover fractions of different LC classes is needed (e.g., Masson et al. 2003; Zeng et al. 2002). In addition, vegetation phenology of deciduous areas is often estimated based on leaf area index (LAI, m^2^/m^2^) which is scaled using remotely sensed time-series of normalized difference vegetation index (NDVI) (e.g., Masson et al. 2003; Zeng et al. 2002). Thus, when assessing spatial patterns of tree cover, in the context of an LSM, a systematic way to intercompare tree cover definitions and to separate coniferous and deciduous tree covers need to be established first. In recent years, numerous processes that are known to impact the Earth system dynamics have been incrementally added to LSMs (see, e.g., Fisher and Koven 2020): representations of soil moisture dynamics, land surface heterogeneity, plant and soil carbon cycling, nitrogen and phosphorus cycling, among others. However, while very detailed processes can be represented in site or regional scale simulations, on a global scale, identifying the locations of different vegetation types is of utmost importance. While there have been attempts to represent forest-age structural properties and to account for forest management effects in a climate model simulation, these LC products and models suffice only for regional analyses (see, e.g., Majasalmi et al. 2018, 2020).

Due to a large number of different classifications (and class definitions), direct comparison of different LC products remains complicated. While the common approach to validate LC datasets is to aggregate the finer spatial resolution data to the lower spatial resolution of the LC dataset to study class bin frequencies and confusion matrices, that does not support developing the classifications and improving class definition thresholds. In addition, as the accuracy of classification may be expected to be the worst with the largest number of classes, and the best for the most simplified classifications, the confusion matrix approach does not allow meaningful intercomparison of the products. Alternatively, the categorical classifications can be transformed into continuous tree cover estimates based on their class definition thresholds (“bin means”). After all products have been converted to continuous estimates of tree cover, different metrics can be used to intercompare the tree covers both statistically and spatially.

The aims of this study are: (1) to assess and intercompare tree cover classifications currently employed by three different global LC product series and (2) to demonstrate an approach which allows a flexible intercomparison of different tree cover representations of the LC products. Our case study is based on the boreal forests in Finland. For the intercomparison of conifer and deciduous tree covers, we analyzed the data after it was aggregated to the spatial resolution employed in climate modeling studies (i.e., using the Climate Modeling Grid).

## Materials and methods

### Study area

We used Finland (area 338,440 km^2^) as the case study area because more than 70% of Finnish land surface area is classified as forest by FAO (FRA FIN 2015) (i.e., ~222,180 km^2^). Finland is located in the Northern European boreal zone area (bounding box: 20.6° E, 31.5° E, 59.8° N, 70.1° N) and Finnish forests have been under intensive forest management for several decades and belong to the most intensively studied forest areas in Europe. Finnish boreal forest is dominated by evergreen conifers Norway spruce (*Picea abies*) and Scots pine (*Pinus sylvestris*). Broad-leaved deciduous tree species Silver birch (*Betula pendula*) and Downy birch (*Betula pubescence*) rarely form “pure” (i.e., single species) forests. Other broad-leaved or coniferous deciduous tree species such as European aspen (*Pupulus tremula*), alders (*Alnus glutinosa*, *Alnus incana*), English oak (*Quercus robur*), Norway maple (*Acer platanoides*), or larches (*Larix* spp.) occasionally occur as a mixed species but never form pure forests. At the landscape level, about 10% of tree cover is deciduous. Thus, in this study, “coniferous” refers to evergreen conifers (i.e., pine and spruce), while “deciduous” is used to include all tree species that shed their leaves for winter (i.e., broad-leaved trees and larches).

### Materials

#### MS-NFI maps

Since 2012, Finnish MS-NFI data has been publicly available online in raster map form. The raster maps are provided for over 40 different forest themes such as dominant tree species, growing stock volume, and biomass at a spatial resolution of 16 m × 16 m. In this study, we used the MS-NFI thematic maps of “canopy cover 2017 (%),” “deciduous canopy cover 2017 (%),”and Lorey’s height (the height of the median tree) of “*H*” (dm). In addition, a mask to extract Finnish land surface area from the global products was prepared using the MS-NFI thematic map of “land class based on FAO FRA.” The uniqueness of the MS-NFI maps comes from the fact that the algorithm has been trained using forest inventory data from 53,989 field plots covering the entirety of Finland.

The MS-NFI 2017 products are available as ready products (©Natural Resources Institute Finland 2019; for further details, see, e.g., Mäkisara et al. (2019)). MS-NFI maps are processed using an improved *k*-nearest-neighbor method in which the weights of the features were sought using an optimization method based on a genetic algorithm. The other land categories, as well as water bodies, were delineated out using the elements of the topographic database of the Land Survey of Finland. The optical satellite images used to create the products included eight Sentinel-2A MSI images, six Sentinel-2B MSI images, and 19 (seven orbits) Landsat 8 OLI images. The original map coordinate system is EPSG 3047 (ETRS-TM35FIN).

The tree cover definition of MS-NFI tree cover maps is “…canopy cover of trees is the vertical projection area on the horizontal plane of the canopies of the individual trees on a field plot.” As canopy cover definition does not separate the contributions of coniferous (evergreen) and deciduous (broadleaved) species, deciduous canopy cover was derived based on the basal area (i.e., the cross-section area of the tree stems of a stand per hectare and measured at a height of 1.3 m (MS-NFI 2019)). According to the MS-NFI (2019) manual: “the canopy cover proportion of broad-leaved trees is derived from the total cover using the basal area. However, in the seedling stands, the canopy cover of broad-leaved trees is assessed using the shares of the stem numbers.” The magnitude of the average error of the estimates of tree cover at pixel level varies between 14 and 20% and average tree height (H, m) between 3.5 and 5.9 m (MS-NFI 2019). It is noteworthy that, although the estimation errors at pixel level are rather high, they tend to decrease as the area of the interest increases and contains more pixels (MS-NFI 2019).

#### Global land cover products

We used annual Collection 6 (C6) MODIS products: (1) land cover products suite of MCD12Q1 and (2) MOD44B vegetation continuous fields (VCF), as well as a land cover product by the European Space Agency Climate Change Initiative (ESA CCI), which is the only non-MODIS-based data product. All products (Table 1) are annual products for the year 2017. VCF is based on regression trees (MODIS VCF User guide 2020), and MCD12Q1 land cover products suite is produced using supervised classification techniques such as decision trees (MODIS MCD12 2020) and ensemble classification methods (Friedl et al. 2002, 2010). The CCI LC is based on machine learning methods that combine supervised and unsupervised algorithms (Poulter et al. 2015).

**Table 1:** Land cover/use products used in this study. Abbreviations: ‘Product’ is the official product name, ‘Name’ is the acronym used to refer to particular dataset, ‘Resolution’ is the original spatial resolution of the product, ‘Type’ determines if labeling is continuous or categorical (i.e. classified)

Product	Product type	Name	Resolution	Type	Reference
MS-NFI	Canopy cover	MS-NFI	16 m	Continuous	Mäkisara et al. 2019
MOD44B	Tree cover	VCF	250 m	Continuous	MODIS VCF 2020
CCI LC	Land cover	CCI	300 m	Classified	Poulter et al. 2015
MCD12Q1	Land cover	IGBP	500 m	Classified	Loveland and Belward 1997
MCD12Q1	Land cover	LAI	500 m	Classified	Myneni et al. 2002
MCD12Q1	Land cover	PFT	500 m	Classified	Bonan et al. 2002
MCD12Q1	Land cover	LCCS1	500 m	Classified	MODIS LCCS 2020
MCD12Q1	Land cover/use	LCCS2	500 m	Classified	MODIS LCCS 2020
MCD12Q1	Land cover/use	LCCS3	500 m	Classified	MODIS LCCS 2020

The MODIS Land Cover Type product (MCD12Q1) contains a suite of science data sets which map land cover globally (and annually) using six different land cover legends. They include five legacy classifications (IGBP, UMD, LAI, BGC, and PFT) and three Land Cover Classification System (LCCS) layers from the FAO (the first is meant for land cover, the second for land use, and third for surface hydrology applications). We used all of these classifications except two: we excluded the BGC classification from this study because the tree height data used in MS-NFI field inventories was measured from a breast-height-diameter of 1.3 m, whereas the BGC product uses a definition of 1 m. In addition, as IGBP and UMD schemes are equal for forest classes, UMD was also excluded from the analyses. The product coordinate reference system (crs) is SR-ORG 6842 (i.e., MODIS Sinusoidal). The legends used by different classifications are available from MODIS MCD12 (2020).

The MOD44B VCF is a yearly product representing global surface vegetation cover as gradations of three ground cover components: percent tree cover, percent non-tree cover, and percent non-vegetated (bare). In this study, only the component “percent tree cover” was used. The VCF product map crs is EPSG 4326.

The CCI annual land cover product (v.2.1.1) follows standardized hierarchical classification by the United Nations Land Cover Classification System (UN-LCCS), which allows conversion from land cover classes into PFTs using a cross-walking table (e.g., Poulter et al. 2015). The LC product legend is available from CCI LCCS (2020). The product was provided in the netcdf file format, and after rasterization was projected to crs of EPSG 4326.

#### Climate Modeling Grid

The low-resolution LC product MCD12C1, also known as the MODIS Climate Modeling Grid (MODIS CMG, i.e., at 0.05° ~ 5.6 km) (MODIS CMG 2020), was used here as a basis for spatially comparing the different medium and high spatial resolution LC product classifications. The MCD12C1 projection is EPSG 4008.

### Processing

#### Preprocessing

Spatial subsets were clipped from the global LC products (i.e., VCF, CCI, and the six MCD12Q1 products) to cover an area defined by the bounding box of Finland. These subsets were projected to Finnish UTM (i.e., EPSG 32635, zone 35) and masked using “land class based on FAO FRA” to include only areas belonging to Finnish land surface area (i.e., excluding land areas of neighboring countries). The MS-NFI tree height and tree cover tiles were mosaicked and projected to Finnish UTM. The coniferous tree cover fraction was obtained by subtracting deciduous tree cover from the total tree cover. The MS-NFI data were resampled to correspond to the LC product extents and resolutions (i.e., VCF, CCI, and MCD12Q1).

#### Assessment of the LC product tree cover classes

To assess the tree cover classifications employed by the global LC products, the MS-NFI data was classified into the LC product classes and only tree cover classes were included (i.e., classes such as LCCS2 class “forest/cropland mosaic” were excluded). Details regarding the classes (for original legend class definitions, see MODIS MCD12 (2020) and CCI LCCS (2020)) and their implementation in our case study are provided in Table 2. It is noteworthy that the VCF and the CCI classifications do not employ any tree height thresholds (i.e., canopy > 2 m) which are used by majority of the other classifications. The default continuous VCF tree cover product was binned into five equally spaced bins to assess the overall classification performance at low and high tree covers (see details regarding the bins in Table 2).

**Table 2:** LC-product tree cover class definitions and details regarding classification implementation of MS-NFI data. Abbreviations: CC = total tree cover, CC_c = conifer tree cover, CC_d = deciduous tree cover. Note, in absence of clear definition for the CCI mixed-class, it was classified using the respective IGBP/LCCS1 mixed-class definition

Classification	Original legend class (and class number) or bin	Details regarding classification implementation
VCF	CC 10	bin(0.01–20)– > CC 10
	CC 30	bin(20.01–40)– > CC 30
	CC 50	bin(40.01–60)– > CC 50
	CC 70	bin(60.01–80)– > CC 70
	CC 90	bin(80.01–100) – > CC 90
IGBP (UMD)	ENF (1)	CC_c > CC_d; CC > 60; *h* > 2
	DNF + DBF (3, 4)	CC_d > CC_c; CC > 60; *h* > 2
	Mixed (5)	CC > 60; CC_c & CC_d = 40–60; *h* > 2
	Woody savannas (8)	30 > = CC < = 60; *h* > 2
	Savannas (9)	10 > = CC < 30; *h* > 2
LAI	EBF + ENF (5,7)	CC > 60; CC_c > CC_d; *h* > 2
	DBF + DNF (6,8)	CC > 60; CC_d > CC_c; *h* > 2
	Savannas (4)	10 > = CC < = 60; *h* > 2
PFT	ENT + EBT (1,2)	CC >10; CC_c > CC_d; *h* > 2
	DNT + DBT (3,4)	CC >10; CC_d > CC_c; *h* > 2
LCCS1	ENF (11)	CC > 60; CC_c > CC_d; *h* > 2
	DNF + DBF (13,14)	CC > 60; CC_d > CC_c; *h* > 2
	Mixed (15,16)	CC > 60; CC_c & CC_d = 40–60; *h* > 2
	Open forest (21)	30 > = CC < = 60; *h* > 2
	Sparse forest (22)	10 > = CC < 30; *h* > 2
LCCS2	Dense forest (10)	CC >60; *h* > 2
	Open forest (20)	10 > = CC < = 60; *h* > 2
LCCS3	Dense forest (10)	CC >60; *h* > 2
	Open forest (20)	10 > = CC < = 60; *h* > 2
CCI	NET (70)	CC_c > 15
	BDT + NDT (60,80)	CC_d > 15
	Mixed (90)	CC > 15; CC_c and CC_d = 40–60

*Footnotes: VCF* vegetation continuous fields, *IGBP* International Geosphere-Biosphere Programme, *UMD* University of Maryland, *ENF* evergreen needleleaf forests, *DNF* deciduous needleleaf forests, *DBF* deciduous broadleaf forests, *LAI* leaf area index, *EBF* evergreen broadleaf forest, *PFT* plant functional types, *ENT* evergreen needleleaf trees, *EBT* evergreen broadleaf trees, *DNT* deciduous needleleaf trees, *DBT* deciduous broadleaf trees, *LCCS1-3* FAO-Land Cover Classification System land cover 1–3, *CCI* climate change initiative, *NET* needleleaved evergreen trees, *BDT* broad-leaved deciduous trees, *NDT* needleleaved deciduous trees

All CCI LC product classes (i.e., NET, BDT, NDT, and mixed; see explanation of acronyms in Table 2) employ a tree cover definition of “>15%.” CCI does not provide a definition for its mixed class and thus, it was classified using the respective IGBP mixed class definition. Additionally, a separate three-step classification was needed to assign the MS-NFI data into the CCI LC product classes, because otherwise nearly all pixels would have been assigned to the most abundant (NET) class. First, all pixels belonging to the mixed-tree cover class were classified into the mixed class. Then, after excluding mixed-classified pixels, all pixels where BDT+NDT tree cover was larger 15% were classified into the BDT+NDT class. Finally, after excluding pixels classified into mixed and BDT+NDT classes, all pixels where NET tree cover was larger than 15% were classified into the NET class. It can be noted that Finland does not have large NDT dominated forest areas, but the global LC products do classify some Finnish land area to belong into NDT class. Thus, we had to assign those pixels either into either the coniferous or deciduous species group. Since NDT tree canopy winter albedo is more similar to that of deciduous trees than that of evergreen conifers, and because winter albedo has a strong impact on the land surface energy balance at high latitudes, NDT was classified as belonging to the deciduous species group.

#### Intercomparison of the tree cover classes in LC products

In order to allow better intercomparison of different LC products and classifications, an approach called a “translation legend” (Table 3) was developed. The idea of a translation legend is to translate each categorical forest class into a numeric “tree cover fraction” using a class mean tree cover value and to separate the tree cover into coniferous and deciduous tree covers following the original LC product legend class definitions. Notably, the number of LC classes that can be included is higher when using an approach based on a translation legend than in classifying MS-NFI data into LC product classes. This is because partially covered tree cover classes (such as LCCS2 class “forest/cropland mosaic”) can be included.

**Table 3:** Translation legend* for intercomparison of land cover/use classifications by converting categorical classes into continuous representations of tree cover. For simplicity, we used a 2 m height threshold (i.e., H-limit-column) for all except the CCI classification. Pixel “total tree (%)” shows the amount of within-pixel tree cover, and “Decid.(%)” and “Conifer (%)” the respective species composition. For classifications that do not separate between species, no separation was attempted

Classification	Original legend class (and class number)	CC limit (%)	*H* limit (m)	Total tree (%)	Decid. (%)	Conifer (%)
IGBP (UMD)	ENF (1)	>60	>2	80		100
	DNF + DBF (3, 4)	>60	>2	80	100	
	Mixed savannas (5)	>60	>2	80	50	50
	Woody savannas (8)	30–60	>2	45	(50)	(50)
	Savannas (9)	10–30	>2	20	(50)	(50)
	Cropland/Natural veg. mosaic (14)	25	(>2)	25	(50)	(50)
LAI	EBF + ENF (5,7)	>60	>2	80		100
	DBF + DNF (6,8)	>60	>2	80	100	
	Savannas (4)	10–60	>2	35	(50)	(50)
PFT	ENT + EBT (1,2)	>10	>2	55		100
	DNF + DBT (3,4)	>10	>2	55	100	
LCCS1	ENF (11)	>60	>2	80		100
	DNF + DBF (13,14)	>60	>2	80	100	
	Mixed (15,16)	>60	>2	80	50	50
	Open forest (21)	30–60	>2	45	(50)	(50)
	Sparse forest (22)	10–30	>2	20	(50)	(50)
LCCS2	Dense forest (10)	>60	>2	80		
	Open forest (20)	10–60	>2	35		
LCCS3	Dense forest (10)	>60	>2	80		
	Open forest (20)	10–60	>2	35		
	Woody wetlands (27)	>10	(>2)	45		
	Tundra (51)	<10	(>2)	5		
CCI	BDT + NDT (60, 80)	>15		57.5	100	
	NET (70)	>15		57.5		100
	Mixed (90)			50	50	50
	Mosaics (30, 40, 100, 110)			25	(50)	(50)
	Sparse vegetation (150, 160)	<15		7.5	(50)	(50)

*Footnotes: *IGBP* International Geosphere-Biosphere Programme, *UMD* University of Maryland, *ENF* evergreen needleleaf forests, *DNF* deciduous needleleaf forests, *DBF* deciduous broadleaf forests, *LAI* leaf area index, *EBF* evergreen broadleaf forest, *PFT* plant functional types, *ENT* evergreen needleleaf trees, *EBT* evergreen broadleaf trees, *DNT* deciduous needleleaf trees, *DBT* deciduous broadleaf trees, *LCCS1-3* FAO-Land Cover Classification System land cover 1–3, *CCI* climate change initiative, *NET* needleleaved evergreen trees, *BDT* broad-leaved deciduous trees, and *NDT* needleleaved deciduous trees

For most classifications, tree cover and vegetation height thresholds were used. The only exception was the CCI product which does not apply a height threshold. The LCCS3 class “Woody wetlands” was the only classification using a height threshold over 1 m (i.e., for all other classifications employing height threshold, it is >2 m) and thus, the >2-m height threshold was used also for that class. Finally, note that as VCF is continuous it does not count as “classification”, despite being a tree cover product.

First, for each LC product pixel, the original legend class was replaced with a mean tree cover estimate as defined by its original legend definition. For example, in the case of the IGBP forest tree cover limit of “>60%” would have 80% mean tree cover (i.e., maximum being 100%), and respectively IGBP woody savanna “30–60%” would have 45% mean tree cover (Table 3). All other classes not listed in Table 3 were assigned a forest tree cover value of zero. The “mosaic” classes (i.e., containing a mixture of LC types) pose challenges, and here, a 25% rule was applied in the absence of better information (e.g., in the case of IGBP and CCI). In other words, 25% of the pixel was assumed to have tree cover. As VCF contains a continuous tree cover fraction, no translations were done. It is noteworthy that we may expect the forest cover values to saturate at around 80% cover due to the applied transition legend values. This is, however, a reasonable assumption in boreal forests, as boreal forest trees tend to have long and narrow crowns and have large gaps between individual crowns, whereas in temperate forests the tree cover may often be close to 100% (e.g., Horn 1971). For all classifications separating coniferous and deciduous tree covers, the classes without species share information were assumed to account for 50% of the total tree cover (e.g., IGBP “Savanna” class note markings “(50)” in Decid. (%) and Conifer (%) columns). For classifications that do not separate between species, no separation was attempted.

#### Spatial aggregation and mapping

The MS-NFI data was first aggregated to correspond to the LC product resolutions (i.e., VCF, CCI, and MCD12Q1) using aggregation factors (i.e., taking the mean of *X*×*X* pixel windows to create larger cells) of 9, 15, and 23 for VCF, CCI, and MCD12Q1, respectively. These factors were obtained after all the data was projected to Finnish UTM by calculating how many of the smaller pixels are needed to fill in one larger pixel and taking the mean of these pixels.

After aggregation, the MS-NFI data was resampled to LC product resolutions (i.e., VCF, CCI, and MCD12Q1) and reclassified into three tree cover bins of 0–30%, 31–60%, and 61–100% in order to assess the classification flexibility to represent areas with high, moderate, and low tree cover.

After the translation legend had been used to convert categorical cover classes to continuous tree covers, all tree cover data was aggregated to MODIS CMG resolution to inspect the spatial pattern in species distributions. Separate coniferous and deciduous tree cover maps were created using aggregation factors of 224 for MS-NFI maps, 15 for CCI, and 9 for MCD12Q1. The resulting maps allowed spatial intercomparison of coniferous and deciduous tree covers at a resolution meaningful for a range of LSMs.

#### Accuracy assessment

The assessment of the LC classifications included analysis of pixel counts belonging to different LC classes and confusion matrices between the LC product classes and MS-NFI data based classes. For the LC product intercomparison, we used confusion matrices, the root mean squared error (RMSE), the mean bias error (MBE), and coefficient of determination (*r*^2^).

The MBE and RMSE are defined as:

1 $$ \mathrm{MBE}=\frac{\sum_{i=1}^n\left({P}_i-{R}_i\right)}{n} $$

2 $$ \mathrm{RMSE}=\sqrt{\frac{\sum_{i=1}^n{\left({P}_i-{R}_i\right)}^2}{n}} $$

where *i* is the pixel index, *P* is the tree cover from the LC products (after applying the translation legend), *R* is the tree cover from the MS-NFI data, and *n* is the sample size. The RMSE and MBE were calculated also for the low, moderate, and high tree cover bins (i.e., at 0–30%, 31–60%, and 61–100%, respectively).

## Results

### Assessment of tree cover estimates in the LC products

Based on pixel counts belonging to different tree cover classes (i.e., ignoring the spatial distribution of those classes), tree cover in Finland was either slightly overestimated or underestimated by different LC products and classifications (Table 4). Compared to the MS-NFI data, underestimation of the tree cover was noted for VCF, PFT, and CCI products (at 6%, 2%, and 11%, respectively). For example, the 11% underestimation observed in the CCI product indicates that 11% of tree-covered areas in Finland were not classified as forest by the CCI product. A slight overestimation of tree-covered areas was noted for most MODIS-based classifications. The overestimation of tree cover was 3% for LAI, LCCS1, and LCCS2, and 2% for IGBP and LCCS2. Thus, the best performing classifications in terms of mapping tree-covered areas were PFT and IGBP, and LCCS3.

**Table 4:** Validation statistics. LC% and MS-NFI% columns contain fractional covers of pixels belonging to different classes, using MS-NFI classified total pixel count as a denominator (other abbreviations are explained in Table 2; note, also LC% column values were divided with MS-NFI total pixel count)

Classification	Original legend class (and class number) or bin		Pixel count, LC	Pixel count, MS-NFI	LC%	MS-NFI%
VCF	CC 10		2,219,830	3,118,988	17.06	23.96
	CC 30		4,389,648	3,181,799	33.73	24.45
	CC 50		4,956,781	4,131,576	38.08	31.74
	CC 70		692,488	2,578,757	5.32	19.81
	CC 90		32	4168	0	0.03
		sums:	12,258,779	13,015,288	94	100
IGBP	ENF (1)		383,769	266,961	18.78	13.07
	DNF + DBF (3, 4)		267	2095	0.01	0.1
	Mixed (5)		291,834	17,942	14.28	0.88
	Woody savannas (8)		1,105,743	1,186,598	54.12	58.07
	Savannas (9)		292,947	569,697	14.34	27.88
		sum:	2,074,560	2,043,293	102	100
LAI	EBF + ENF (5, 7)		673,910	279,957	32.98	13.7
	DBF + DNF (6, 8)		5687	7041	0.28	0.34
	Savannas (4)		1,429,597	1,756,295	69.97	85.95
		sum:	2,109,194	2,043,293	103	100
PFT	ENT + EBT (1, 2)		1,988,314	1,923,139	97.31	94.12
	DNT + DBT (3, 4)		19,637	120,109	0.96	5.88
		sum:	2,007,951	2,043,248	98	100
LCCS1	ENF (11)		387,342	266,961	18.96	13.07
	DNF + DBF (13,14)		285	2095	0.01	0.1
	Mixed (15,16)		292,003	17,942	14.29	0.88
	Open forest (21)		1,130,093	1,186,598	55.31	58.07
	Sparse forest (22)		303,059	569,697	14.83	27.88
		sum:	2,112,782	2,043,293	103	100
LCCS2	Dense forest (10)		679,597	286,998	33.26	14.05
	Open forest (20)		1,429,597	1,756,295	69.97	85.95
		sum:	2,109,194	2,043,293	103	100
LCCS3	Dense forest (10)		675,903	286,998	33.08	14.05
	Open forest (20)		1,402,234	1,756,295	68.63	85.95
		sum:	2,078,137	2,043,293	102	100
CCI	NET (70)		3,229,056	3,413,809	74.37	78.63
	BDT + NDT (60,80)		90,648	543,029	2.09	12.51
	Mixed (90)		525,994	384,789	12.12	8.86
		sum:	3,845,698	4,341,627	89	100

In terms of classification accuracy (i.e., accounting for the spatial locations of pixels belonging to different classes), the poorest performance was observed, as expected, for classification with the largest number of classes (i.e., IGBP and LCCS1) (Table 5). The accuracy of these two classifications was approximately 42%. The continuous VCF product, which was reclassified into five bins to analyze the overall classification performance, performed equally poorly based on its classification accuracy. The CCI (49%), and LAI, LCC2, and LCCS3 classifications (each ~60%) were more accurate then IGBP, LCCS1, and VCF classifications (each ~42%) (percentage of accurately classified pixels in parenthesis). Note that LCC2 and LCCS3 employed the same classification (Table 2), but had differences in spatial mapping of class “open forest.” The highest accuracy was observed for the classification PFT (accuracy was ~75%) as it had only two classes.

**Table 5:**
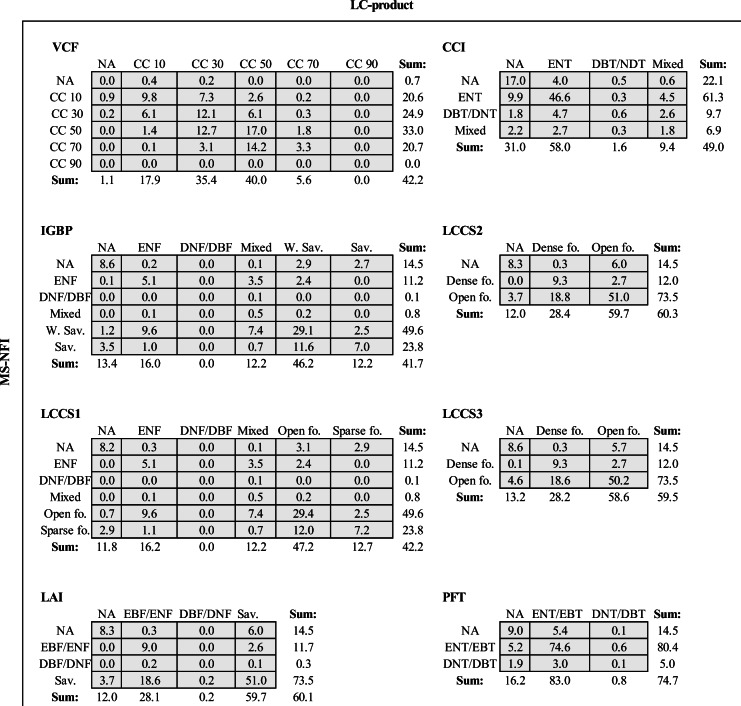
Confusion matrix between different tree cover classes (or bins) at LC product original resolution. NA = no tree cover, W. Sav. = Woody savannas, Sav. = Savannas, Open fo. = Open forest, Sparse fo. = Sparse forest, Dense fo. = Dense forest (see Table 2 for other abbreviations)

VCF underestimated most areas with low and high tree covers (i.e., bins “CC 10” and “CC 70”), while it overestimated the area belonging to the two intermediate bins (i.e., “CC 30” and “CC 50”) (Table 5). For IGBP and LCCS1 (which employ the same classification but have differences in LC products), the poorest performing class was mixed, which contained approximately 12% of pixels but based on MS-NFI data, only 0.8% of pixels belonged to that class. Deciduous tree cover classification results of IGBP, LCCS1, and LAI were similar due to identical class definitions (Table 2). According to Table 5, the most accurate single-class classifications were woody savanna for IBGP (29.1%), savanna for LAI (51%), and open forest for LCCS1 (29.4%). The CCI map product only correctly detected 0.6% of the deciduous tree cover whereas the respective MS-NFI-based estimate was 10% (Table 5). The finer spatial resolution of the CCI product compared to MCD12Q1 classifications (Table 1) clearly improved mapping deciduous tree cover.

### Intercomparison of tree cover estimates in the LC products

Tree cover estimates derived from the LC products using the transition legend were almost always systematically higher than the tree cover values obtained from the MS-NFI data (Table 6). Using all data, the classification with the smallest deviation from the reference MS-NFI values was VCF (RMSE of 16.7% and MBE of −3.4), closely followed by CCI (RMSE of 20.4 and MBE of 9.6) (Table 6). As could be expected, for all MCD12Q1 based classifications, almost equal RMSE values (“RMSE all”—column in Table 6) were obtained using different classifications. However, in general, the tree cover representation was more successful in areas with high tree cover than in areas with low tree cover.

**Table 6:** Intercomparison of the total tree cover and binned tree covers (All = all tree cover data after application of the translation legend, Low = tree cover bin of 0–30%, Moderate = tree cover bin of 31–60%, and High = tree cover bin of 61–100%). Abbreviations: MBE = mean bias error, RMSE = root mean squared error, and r^2^ = coefficient of determination. Statistics were calculated using the original LC–product resolutions

Tree cover product	MBE	MBE	MBE	MBE	RMSE	RMSE	RMSE	RMSE	*R*^2^
	Low	Moderate	High	All	Low	Moderate	High	All	All
VCF	9.0	−6.5	−15.5	−3.4	18.4	14.4	18.6	16.7	0.4
CCI	28.5	8.3	−10.4	9.6	32.7	14.4	13.0	20.4	0.1
IGBP	20.8	10.2	7.4	13.0	27.3	19.9	16.5	22.0	0.3
LAI	20.9	4.9	5.2	9.9	25.3	20.8	19.3	22.1	0.3
PFT	37.4	9.3	−9.7	15.4	38.4	12.6	10.3	23.7	NA
LCCS1	21.0	10.1	7.4	13.1	27.5	19.9	16.5	22.2	0.3
LCCS2	20.9	4.9	5.2	9.9	25.3	20.8	19.3	22.1	0.3
LCCS3	21.0	4.9	5.2	10.0	25.4	20.7	19.3	22.1	0.3

For VCF, almost equal RMSE values were noted for the highest and lowest tree cover bins, whereas for all other classifications, the general pattern was that the largest RMSE values occurred in the lowest tree cover bin and got smaller towards higher tree cover bins (Table 6). For VCF, a positive bias was noted for the lowest tree cover bin which became increasingly negative towards higher tree covers. In general, positive biases were noted for both low and moderate tree cover bins, as well as some negative biases for the highest tree cover bin (Table 6). The most linear relationship between the tree cover derived from the LC products and the MS-NFI data was observed for VCF. As linearity results from having more tree cover classes with (possibly) different mean-tree cover values, it can be used to assess classification skills to represent variation in tree cover values. In other words, the low *r*^2^ (Table 6) values indicate that there is little variation in tree cover values between coarse spatial resolution LC product pixels compared to that represented by MS-NFI data.

Confusion matrices were used to illustrate some deficiencies in classification performance. For example, CCI, PFT, and LCCS2-based tree cover estimates are not able to represent areas belonging to the highest tree cover bin (Table 7). PFT and LAI classifications, on the other hand, suffer from misrepresentation of areas belonging to the lowest tree cover bin. In MS-NFI data, areas with moderate tree cover were the most abundant (i.e., 46–56% cover, note that range is provided as original LC product resolutions were used to calculate the statistics), followed by areas belonging to the lowest tree cover bin (i.e., 25–32%) and to the highest tree cover bin (i.e., 14–22%) (Table 7). In terms of a classification’s ability to represent low, moderate, and high tree covers, the most flexible classifications were VCF, IGBP, LCCS1, and LCCS3 (Table 7).

**Table 7:**
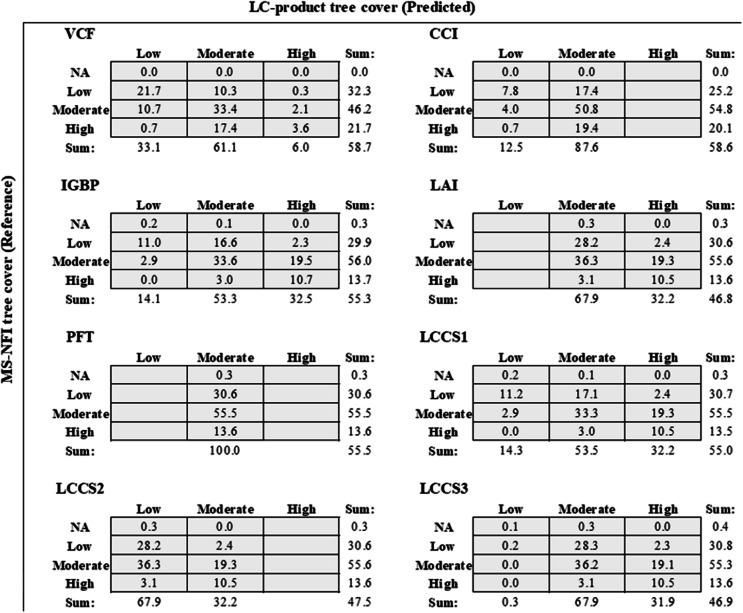
Confusion matrix between different within pixel tree covers at LC–product original resolution. NA = no tree cover, Low = pixel tree cover bin of 0–30%, Moderate = pixel tree cover bin of 31–60%, and High = pixel tree cover bin of 61–100%. Accuracy is the sum of correctly classified pixels (diagonal sum, excluding the first NA–row)

### Intercomparison of coniferous and deciduous tree cover

Coniferous and deciduous tree cover estimates derived from the LC products using the transition legend showed clear differences in error values between the two species groups (Table 8). The deviations from the reference MS-NFI values were, in general, higher for coniferous species compared to deciduous species. However, the highest RMSE among all classifications was observed for the deciduous PFT classification (Table 8). For coniferous tree covers, the smallest RMSEs and biases were noted in the IGBP and LCCS1 classifications, which had the largest number of tree cover classes. For deciduous tree covers, the smallest RMSE was in the LAI classification which only used three tree cover classes. For deciduous species, positive biases were noted for the lowest tree cover bins, whereas negative biases were observed for moderate or high tree cover bins (except for PFT) (Table 8). For coniferous species, positive biases were also observed for the lowest tree cover bins but for the moderate or high tree cover bins, the biases were either positive or negative depending on the classification.

**Table 8:** Intercomparison of the coniferous and deciduous tree covers and binned tree covers with the reference tree cover estimates from MS-NFI (*All* all tree cover data after application of the translation legend, *Low* tree cover bin of 0–30%, *Moderate* tree cover bin of 31–60%, *High* tree cover bin of 61–100%). Abbreviations: *MBE* mean bias error, *RMSE* root mean squared error, *r*^*2*^ coefficient of determination. Statistics were calculated using the original LC product resolutions

Tree cover product	MBE	MBE	MBE	MBE	RMSE	RMSE	RMSE	RMSE
	Low	Moderate	High	All	Low	Moderate	High	All
Coniferous								
CCI	24.5	7.5	−9.0	13.4	31.6	17.3	14.8	23.6
IGBP	6.5	−2.2	3.4	1.7	18.4	23.5	22.6	21.4
LAI	9.4	3.5	10.0	6.2	23.5	29.6	22.9	27.0
PFT	37.6	12.0	−7.2	22.5	38.6	14.3	7.4	27.2
LCCS1	6.6	−2.2	3.3	1.8	18.5	23.5	22.7	21.4
Deciduous								
CCI	10.1	−8.9	−29.4	9.1	16.1	17.5	34.4	16.1
IGBP	15.5	−2.3	−26.5	15.3	17.5	10.9	27.4	17.5
LAI	11.9	−9.8	−8.1	11.8	13.5	21.8	30.8	13.5
PFT	45.7	19.3	−8.7	45.0	46.2	20.0	8.8	45.7
LCCS1	15.5	−2.5	−26.5	15.3	17.5	11.0	27.4	17.4

### Intercomparison of coniferous and deciduous tree covers using CMG grid

**Fig. 1:**
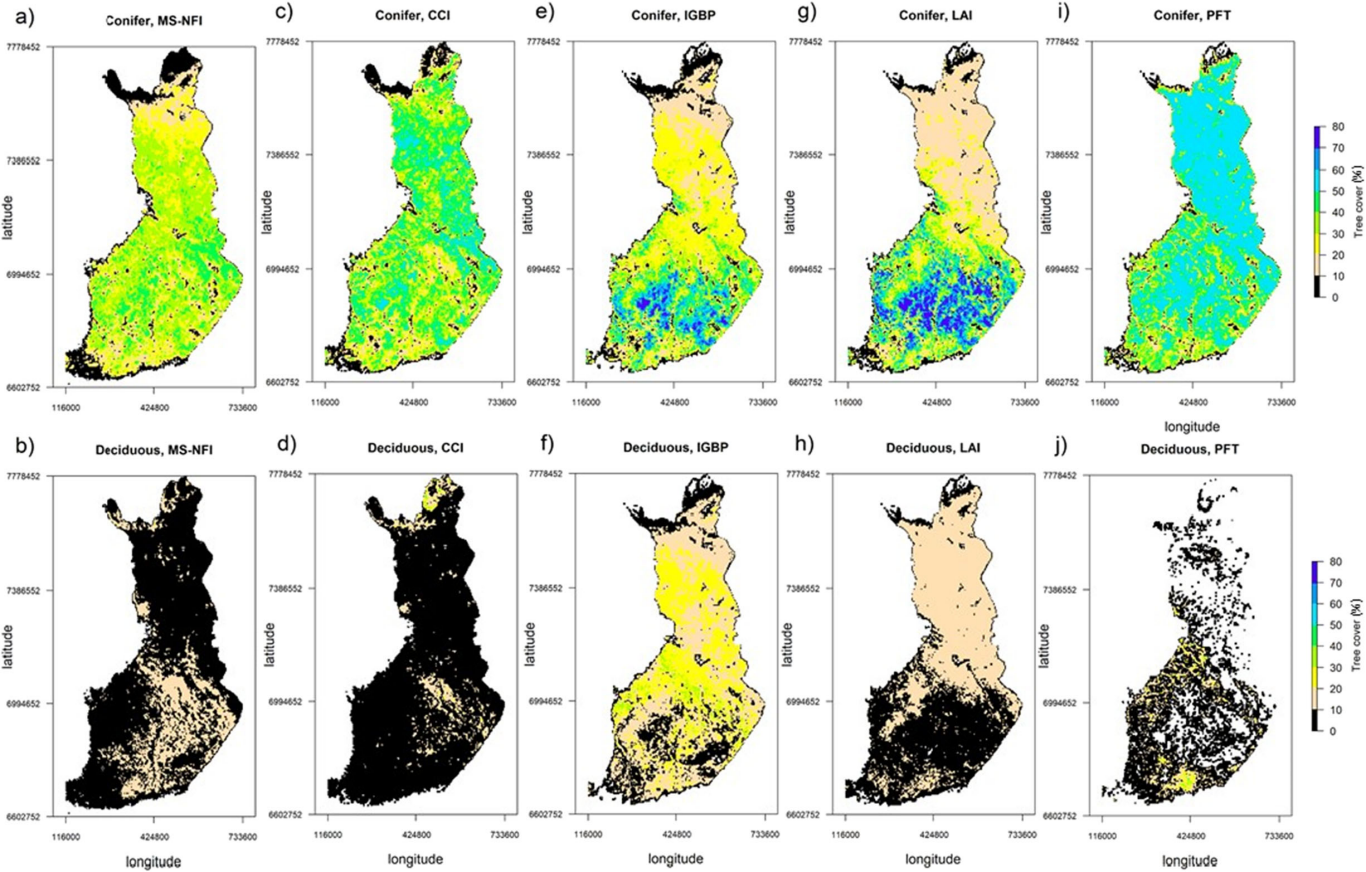
Spatial distribution of coniferous and deciduous tree covers based on four different global LC products and reference data from the Finnish MS-NFI. All data was aggregated and resampled to MODIS Climate Modeling Grid (CMG) resolution. The top row (**a**, **c**, **e**, **g**, **i**) shows tree cover values for coniferous and the lower row (**b**, **d**, **f**, **h**, **j**) for deciduous species. Note: black color is used to denote areas below the 10% CC threshold employed by the international forest definition by FAO

Intercomparison of the maps (Fig. 1) revealed clear differences in spatial patterns of the coniferous and deciduous tree covers. At CMG resolution, the MS-NFI-based coniferous tree cover (Fig. 1a) varied between 20 and 40%, being less than 10% (which is the threshold used by the FAO forest definition) in only some parts of the country. However, the coniferous tree cover maps based on the IGBP, LAI, and PFT classifications based (Fig. 1e, g, i) showed too high or low coniferous tree cover values for large areas. The CCI classification-based coniferous tree cover map (Fig. 1c) appeared the most similar to the MS-NFI-based map—the tree cover values were fairly similar to those of the MS-NFI map, and the CCI map does not show any anomalies in spatial distributions of tree covers, which are present in other maps. For deciduous tree cover, the MS-NFI-based tree cover (Fig. 1b) was often less than 10%, and, for some regions, varied between 10 and 20%. For the IGBP, LAI, and PFT classifications (Fig. 1f, h, j), some clear spatial patterns were observed: all three LC classification-based maps showed either far too high deciduous tree cover values for large areas and/or no deciduous tree cover values. The CCI classification-based map of deciduous tree cover (Fig. 1b) showed the smallest spatial variations in deciduous tree cover values and was also the most similar to the MS-NFI map of deciduous tree cover. These observed differences in the spatial mapping of the tree cover values demonstrate the need to assess also spatial variations present in the map data.

## Discussion

Today’s LC products are often created based on polar-orbiting satellite sensor data. As the areas to be mapped are large, this poses challenges in terms of defining land cover classes to be separated from the satellite sensor data (e.g., Ustin and Gamon 2010). However, as the spatial resolution of the observation unit becomes coarser, the probability of mixed pixels (i.e., different LC classes) increases, which further reduces the number of (forest) classes that have separable spectral and structural properties. An obvious solution would be to achieve a better separation of different LC classes (i.e., forest types) by using higher spatial resolution satellite sensor data from Landsat 8 (L8) and Sentinel 2 (S2), for example. Alternatively, satellite-borne (e.g., GEDI or ICESat-2) or airborne laser scanning data (e.g., from national land surveys) could be used to delineate areas with tree cover and quantify their structural properties (such as canopy cover and leaf area index, e.g., Korhonen et al. 2011; Majasalmi et al. 2017). However, as satellite or airborne laser scanning data is not necessarily free nor readily available for large areas, utilizing optical satellite data remains currently the only operationally feasible solution for tree cover mapping in regional or global applications. Although the higher spatial resolution L8 and S2 data would solve some of the problems associated with low spatial resolution “mixed” pixels, ambiguity would still remain surrounding the “forest” or “tree cover” definitions.

The international forest definition by FAO does not allow direct quantification from space. The obvious problems in applying the FAO forest definition in classifying tree cover from satellite data result from expectations regarding forest life cycle and future development trajectories. In addition, the strict application of the FAO forest classification would mean excluding northern tundra (i.e., dominated by stunted deciduous trees) from forest area, although the growth of these forest areas (e.g., Heiskanen 2008) may be expected to increase due to climate change. Thus, there might be room for redefining “forest” in a way that would allow better mapping of forested areas using remote sensing data, as well as applying the maps as an input for different regional LSM modeling frameworks dealing with forest management or land surface hydrology. Such classification should ideally be based on thresholds that can be retrieved from optical satellite data, and other globally available auxiliary data. A systematic forest definition that allows measuring and monitoring from space is needed for the global quantification of forests, and benchmarking of the national/regional estimates.

In the boreal region, separation between coniferous and deciduous species groups is essential, for example, for better quantification of seasonal courses of surface fluxes in LSMs. In this study, an assessment and intercomparison of today’s most used LC products was conducted, and a new approach, called a translation legend, was developed and applied. This was necessary as there is very limited information available regarding the algorithms (and data) which are used to produce these global LC products. Thus, direct methodological comparisons are not possible. In addition, since each LC product classification uses its own class definitions, there are very few approaches available for validating or intercomparing the informational content of the products.

Application of the translation legend allows systematic intercomparison between different categorical tree cover (i.e., “forest type”) classifications and analysis of spatial distributions of conifer and deciduous species. While traditional LC product assessments based on pixel counts belonging to different classes and incorrectly classified pixels are important, they do not allow developing better LC classifications and class definitions. Application of the translation legend allows converting categorical classes into continuous tree cover values while respecting the original LC class definitions, and intercomparison of either total, or conifer and deciduous tree covers, in a variety of different ways, which provides new insight for product developers and users.

However, as the transition legend is based on converting categorical LC classes into continuous by using bin means (i.e., the true LC distribution that was used to prepare the LC product remains unknown), using a translation legend does not replace the need for traditional LC product validation studies. Rather, it is an extension of conventional validation exercises, a systematic intercomparison tool. The transition legend allows the calculation of statistics (such as *R*^2^, RMSE, MBE) and reclassification of the continuous values for further analysis. In this study, we reclassified the data into three groups of low, moderate, and high tree cover to observe which classifications are flexible enough to represent these variations in the tree cover values. Although the MBEs and RMSEs obtained using the translation legend are not the truth as such, they reflect the classification skill to map different tree cover values. Due to regional differences in conifer and deciduous tree species distributions, we acknowledge the difficulty in developing classifications that would suit all geographical areas.

It is noteworthy that the number of LC classes that can be included in LC product analyses is higher when using the translation legend approach than in traditional assessment because partially covered tree cover classes, such as the LCCS2 class “forest/cropland mosaic,” can be included using the translation legend. This can be accomplished by assigning a partially forested pixel’s respective tree cover fraction (e.g., 25%) adapted from the original legend class definition, and by assuming the share of conifer and deciduous tree species groups is equal. The assumption of equal shares of conifers and deciduous groups of these mixed classes is justified by our study region, as the surroundings of the cultivated areas are often outside the most active forest management operations (i.e., deciduous trees are not harvested as often). In addition, locations close to cultivated areas have a good supply of light, water, and nutrients, all of which benefit the growth of deciduous tree species. Although the area belonging to these partially forested pixels is small in forested landscapes such as in Finland (i.e., forests are fragmented by differently structured forests rather than different LC classes), in other geographical areas fragmented by agriculture and forestry, the fraction of pixels belonging to these mixed classes may be significant. Thus, mixed pixels should be accounted for in tree cover assessments.

As the VCF is continuous by default and the finest spatial resolution tree cover product, it managed to describe variations in tree cover values well. However, it does not separate between conifer and deciduous species, which is an obvious limitation. The CCI product was found to perform well in representing areas with low, moderate, and high tree covers and in separating coniferous and deciduous areas, especially at the final stage in which data was aggregated to the CMG grid resolution. Notably, it is the only classification that did not employ a tree height threshold. Thus, the necessity of the height threshold in forest LC classification may be questioned; we acknowledge that while there is a clear need for a height threshold in forest field inventories, it is perhaps not necessary in global LC classifications, especially as tree height is challenging to retrieve from optical satellite data.

As the LC product maps are independent of the class definitions (i.e., the same classification can be used by several LC products with differences in their spatial class distributions), both must be assessed to evaluate classification performance. For example, in LSMs (or more generally in climate modeling), the climate data (e.g., maps of temperature, and precipitation) are provided as maps and thus, the spatial patterns of coniferous and deciduous tree covers must also be sufficiently mapped to predict vegetation fluxes correctly. Especially in a boreal region, the separation of coniferous and deciduous tree cover areas is necessary due to large effects of tree phenology and snow on surface albedo (Bright et al. 2018).

Direct evaluation of the impacts of varying tree cover descriptions in a climate model requires climate simulations due to the simultaneous usage of different LC products. For example, the community land surface model (CLM) uses MODIS-based monthly mean leaf area index data and IGBP-based fractional covers in its simulations (Zeng et al. 2002). More precisely, while CLM employs IGBP classification to derive vegetation fractional cover values, it uses six alternative biomes (grasses and cereal crops, shrubs, broadleaf crops, savanna, broadleaf forests, and needle forests) defined based on vegetation structure to derive the monthly mean leaf area index values (Myneni et al. 1997). As a result, the vegetation cover fraction and LAI are not constant for each pixel (Zeng et al. 2002) and thus, the impact of fractional cover (i.e., LC product) on climate simulation outcomes cannot be directly evaluated. More attention should be paid to LC data (and therefore also the underlying forest class definitions) that are being used to parametrize the LSMs, as the expected improvements in predicted surface fluxes will rely on the quality of the employed tree cover mapping (e.g., Bright et al. 2018; Majasalmi et al. 2018; Majasalmi et al. 2020).

## Conclusions

We used Finnish MS-NFI data to assess tree cover representations of eight annual global LC classifications for the year 2017 and developed and applied a translation legend approach for better intercomparison of their tree cover representations. The benefits of the developed approach are that it is transparent to apply, can be adapted to any classification and across any spatial scale, and allows the calculation of different statistical metrics. We observed large differences in classification skills of representing variations in tree cover values, and in their spatial mapping of conifer and deciduous tree covers. Based on our analyses, the tree cover was either overestimated or underestimated depending on the LC product, and classification accuracy varied between 42 and 75%. Intercomparison of the LC products revealed clear differences in spatial distributions of conifer and deciduous species. In general, the CCI LC product had the most realistic spatial mapping of coniferous and deciduous tree covers compared to the reference MS-NFI data. As the differences in tree cover mapping may be expected to translate into differences in predicted surface fluxes, users and developers of the LSMs relying on prescribed land cover information are encouraged to pay attention to what type of LC product and classification their analysis is based on. Ideally, the next generation of LC products will be based on a forest definition that facilitates measuring and monitoring from space, and classification that accurately represents coniferous and deciduous species tree covers.

## Data Availability

All data is publicly available (see Table 1, and reference list).

## Material availability

All data is publicly available (see Table 1, and reference list).

## Code availability

Code is available from the author.

## Declarations

### Disclaimer

The text reflects only the authors’ view and the Agency is not responsible for any use that may be made of the information it contains.

## References

[CR1] Bartholome E, Belward AS (2005). GLC2000: a new approach to global land cover mapping from Earth observation data. International Journal of Remote Sensing.

[CR2] Bastin JF, Finegold Y, Garcia C, Mollicone D, Rezende M, Routh D (2019). The global tree restoration potential. Science.

[CR3] Bonan GB, Levis S, Kergoat L, Oleson KW (2002). Landscapes as patches of plant functional types: an integrating concept for climate and ecosystem models. Global Biogeochemical Cycles.

[CR4] Bright RM, Eisner S, Lund MT, Majasalmi T, Myhre G, Astrup R (2018). Inferring surface albedo prediction error linked to forest structure at high latitudes. Journal of Geophysical Research: Atmospheres.

[CR5] CCI LCCS, (2020). https://maps.elie.ucl.ac.be/CCI/viewer/download/CCI-LC_Maps_Legend.pdf. Accessed 10 Jan 2020.

[CR6] Chen JM, Rich PM, Gower ST, Norman JM, Plummer S (1997). Leaf area index of boreal forests: theory, techniques, and measurements. Journal of Geophysical Research: Atmospheres.

[CR7] Fisher RA, Koven CD (2020). Perspectives on the future of Land Surface Models and the challenges of representing complex terrestrial systems. Journal of Advances in Modeling Earth Systems.

[CR8] FRA, (2015). Forest Resource Assessment working paper 180, http://www.fao.org/3/ap862e/ap862e00.pdf. Accessed 10 Feb 2020.

[CR9] FRA FIN, (2015). Global forest Resources Assessment 2015. Country report Finland. Available: http://www.fao.org/3/a-az213e.pdf. Accessed 10 Feb 2020.

[CR10] Friedl MA, McIver DK, Hodges JC, Zhang XY, Muchoney D, Strahler AH (2002). Global land cover mapping from MODIS: algorithms and early results. Remote sensing of Environment.

[CR11] Friedl MA, Sulla-Menashe D, Tan B, Schneider A, Ramankutty N, Sibley A, Huang X (2010). MODIS Collection 5 global land cover: algorithm refinements and characterization of new datasets. Remote Sensing of Environment.

[CR12] Gower ST, Kucharik CJ, Norman JM (1999). Direct and indirect estimation of leaf area index, fAPAR, and net primary production of terrestrial ecosystems. Remote sensing of environment.

[CR13] Gschwantner T, Schadauer K, Vidal C, Lanz A, Tomppo E, Di Cosmo L (2009). Common tree definitions for national forest inventories in Europe. Silva Fennica.

[CR14] Hansen MC, DeFries RS, Townshend JR, Sohlberg R (2000). Global land cover classification at 1 km spatial resolution using a classification tree approach. International journal of remote sensing.

[CR15] Hansen MC, DeFries RS, Townshend JRG, Carroll M, Dimiceli C, Sohlberg RA (2003). Global percent tree cover at a spatial resolution of 500 meters: first results of the MODIS vegetation continuous fields algorithm. Earth Interactions.

[CR16] Hansen MC, Potapov PV, Moore R, Hancher M, Turubanova SAA, Tyukavina A (2013). High-resolution global maps of 21st-century forest cover change. Science.

[CR17] Heiskanen J (2008). Evaluation of global land cover data sets over the tundra–taiga transition zone in northernmost Finland. International Journal of Remote Sensing.

[CR18] Horn, H. S. (1971). *Adaptive geometry of trees (MPB-3)*. *Princeton University Press*. isbn:0-691-08089-5.

[CR19] Jennings SB, Brown ND, Sheil D (1999). Assessing forest canopies and understorey illumination: canopy closure, canopy cover and other measures. Forestry: An International Journal of Forest Research.

[CR20] Korhonen L, Korpela I, Heiskanen J, Maltamo M (2011). Airborne discrete-return LIDAR data in the estimation of vertical canopy cover, angular canopy closure and leaf area index. Remote Sensing of Environment.

[CR21] Loveland TR, Belward AS (1997). The international geosphere biosphere programme data and information system global land cover data set (DISCover). Acta Astronautica.

[CR22] Loveland TR, Reed BC, Brown JF, Ohlen DO, Zhu Z, Yang LWMJ, Merchant JW (2000). Development of a global land cover characteristics database and IGBP DISCover from 1 km AVHRR data. International Journal of Remote Sensing.

[CR23] LUKE, (2020). https://www.luke.fi/tietoa-luonnonvaroista/metsa/metsien-monimuotoisuus/operaatio-mustikka/. Accessed 8 Dec 2020.

[CR24] Majasalmi T, Korhonen L, Korpela I, Vauhkonen J (2017). Application of 3D triangulations of airborne laser scanning data to estimate boreal forest leaf area index. International journal of applied earth observation and geoinformation.

[CR25] Majasalmi, T., Eisner, S., Astrup, R. A., Fridman, J., & Bright, R. M. (2018). An enhanced forest classification scheme for modeling vegetation–climate interactions based on national forest inventory data. *Biogeosciences,15 (2),* 399-412.

[CR26] Majasalmi T, Allen M, Antón-Fernández C, Astrup R, Bright RM (2020). A simple grid-based framework for simulating forest structural trajectories linked to transient forest management scenarios in Fennoscandia. Climatic Change.

[CR27] Mäkisara, K., Katila, M. & Peräsaari, J. (2019). The Multi-Source National Forest Inventory of Finland – methods and results 2015. Natural resources and bioeconomy studies 8/2019, Natural Resources Institute Finland. 57 p. http://urn.fi/URN:ISBN:978-952-326-711-4http://jukuri.luke.fi/handle/10024/543826. Accessed 15 Oct 2019.

[CR28] Masson V, Champeaux JL, Chauvin F, Meriguet C, Lacaze R (2003). A global database of land surface parameters at 1-km resolution in meteorological and climate models. Journal of climate.

[CR29] MODIS CMG, (2020). https://lpdaac.usgs.gov/products/mcd12c1v006/. Accessed 10 Feb 2020.

[CR30] MODIS LCCS, (2020). https://lpdaac.usgs.gov/products/mcd12q1v006/. Accessed 23 Jan 2020.

[CR31] MODIS MCD12, (2020). User guide to collection 6 MODIS Land Cover (MCD12Q1 and MCD12C1) Product. Sulla-Menashe D,, & Friedl M.A. https://modis.ornl.gov/documentation/guides/MCD12_User_Guide_V6.pdf. Accessed 23 Jan 2020.

[CR32] MODIS VCF, (2017). The MOD44B Version 6 Vegetation Continuous Fields (VCF). https://lpdaac.usgs.gov/products/mod44bv006/. Accessed 10 Feb 2020.

[CR33] MODIS VCF, (2020). https://modis-land.gsfc.nasa.gov/vcc.html. Accessed 23 Jan 2020.

[CR34] MODIS VCF User guide, (2020). User Guide for the MODIS vegetation continuous fields product Collection 6, version 1. https://lpdaac.usgs.gov/documents/112/MOD44B_User_Guide_V6.pdf. Accessed 8 Dec 2020.

[CR35] Moon M, Zhang X, Henebry GM, Liu L, Gray JM, Melaas EK, Friedl MA (2019). Long-term continuity in land surface phenology measurements: a comparative assessment of the MODIS land cover dynamics and VIIRS land surface phenology products. Remote sensing of environment.

[CR36] MS-NFI (2019). Multi-source national forest inventory (MS-NFI) raster maps of 2017.

[CR37] Myneni RB, Ramakrishna R, Nemani R, Running SW (1997). Estimation of global leaf area index and absorbed PAR using radiative transfer models. IEEE Transactions on Geoscience and remote sensing.

[CR38] Myneni RB, Hoffman S, Knyazikhin Y, Privette JL, Glassy J, Tian Y (2002). Global products of vegetation leaf area and fraction absorbed PAR from year one of MODIS data. Remote sensing of environment.

[CR39] Poulter B, MacBean N, Hartley A, Khlystova I, Arino O, Betts R (2015). Plant functional type classification for earth system models: results from the European Space Agency’s Land Cover Climate Change Initiative. Geoscientific Model Development.

[CR40] Rautiainen M, Stenberg P, Nilson T (2005). Estimating canopy cover in Scots pine stands. Silva Fennica.

[CR41] Sexton JO, Song XP, Feng M, Noojipady P, Anand A, Huang C (2013). Global, 30-m resolution continuous fields of tree cover: Landsat-based rescaling of MODIS vegetation continuous fields with lidar-based estimates of error. International Journal of Digital Earth.

[CR42] Sexton JO, Noojipady P, Song XP, Feng M, Song DX, Kim DH (2016). Conservation policy and the measurement of forests. Nature Climate Change.

[CR43] Song XP, Huang C, Feng M, Sexton JO, Channan S, Townshend JR (2014). Integrating global land cover products for improved forest cover characterization: an application in North America. International Journal of Digital Earth.

[CR44] Tang H, Song XP, Zhao FA, Strahler AH, Schaaf CL, Goetz S (2019). Definition and measurement of tree cover: a comparative analysis of field-, lidar-and landsat-based tree cover estimations in the Sierra national forests, USA. Agricultural and forest meteorology.

[CR45] Tomppo E, Gschwantner T, Lawrence M, McRoberts RE, Gabler K, Schadauer K (2010). National forest inventories. Pathways for Common Reporting. European Science Foundation.

[CR46] Ustin SL, Gamon JA (2010). Remote sensing of plant functional types. New Phytologist.

[CR47] Zeng X, Shaikh M, Dai Y, Dickinson RE, Myneni R (2002). Coupling of the Common Land Model to the NCAR Community Climate Model. Journal of Climate.

